# Vertebral Column Metastases from an Esthesioneuroblastoma: Chemotherapy, Radiation, and Resection for Recurrence with 15-Year Followup

**DOI:** 10.1155/2013/107315

**Published:** 2013-02-19

**Authors:** Ali S. Shirzadi, Doniel G. Drazin, Allison S. Strickland, Serguei I. Bannykh, J. Patrick Johnson

**Affiliations:** ^1^Department of Neurosurgery, Cedars-Sinai Medical Center, Los Angeles, CA 90048, USA; ^2^Department of Neurosurgery, University of Oklahoma, Oklahoma City, OK 73104, USA; ^3^Department of Pathology and Laboratory Medicine, Cedars-Sinai Medical Center, Los Angeles, CA, USA; ^4^Spine Center, Cedars-Sinai Medical Center, Los Angeles, CA 90048, USA

## Abstract

Esthesioneuroblastoma (ENB) is an uncommon aggressive malignant intranasal neoplasm that originates from neural crest cells of the olfactory epithelium. Although local invasion to the sinuses is common, spinal metastasis of ENB is rare with only 28 documented cases involving the spine spinal cord, or leptomeninges. We report a case of ENB with multiple drop metastases to the cervical and thoracic spine, and review the patient's disease, medical history, and multiple interventions during a span of 15 years following the initial cranial resection. Despite aggressive multiple surgical resections, radiation, and chemotherapy, the tumor had significant progression and recurrence. The literature is reviewed, followed by a discussion of the natural progression of the disease and various reported interventions. Although a combination of surgery with chemotherapy and radiation therapy has been recommended, no definitive management has been established for ENB. Further research is needed to determine decisive treatment for metastatic ENB to the spine.

## 1. Introduction

Esthesioneuroblastoma (ENB), also known as olfactory neuroblastoma, is an uncommon aggressive primitive neuroectodermal malignant neoplasm that arises from olfactory epithelium cells of the upper nasal cavity [1]. ENB is noted in 4 out of 10 million individuals, accounting for 5% of sinonasal and 3% of intracranial tumors [2]. It can present in a wide range of age groups; however, it is mostly noted in a bimodal distribution, occurring most frequently in the second and sixth decades of life [3–5].

ENB tumors display varying clinical behaviors ranging from indolent growth to highly aggressive invasion [6]. An appropriate treatment plan for ENB has not been outlined due to its rarity and lack of control trials; however, surgical resection has been considered as the primary form of treatment alone or in conjunction with radiation therapy and/or chemotherapy [7–9].

Unfortunately, despite aggressive therapy, ENB has been noted to have a high local recurrence rate of 50–60% with 10–62% presenting as metastatic cases and 20–30% of those cases involving the CNS [7, 10]. Once ENB cells invade the cribriform plate, they can spread to the anterior skull base, extend to the brain parenchyma or leptomeninges and can lead to drop metastasis anywhere in central neural axis [7, 11, 12]. CNS metastasis is usually noted 0–10 years after the initial diagnosis and reported as having a survival expectancy of 2 years or less [13]. Spinal metastasis of ENB is rare with only 28 documented cases involving the spine, spinal cord, or leptomeninges. We report a case of ENB with multiple drop metastasis and significant progression of the tumor despite multiple aggressive surgical resections in conjunction with chemotherapy and radiation therapy.

## 2. Case Report

A 54-year-old male physician presented to the emergency department with a 4-day duration complaint of ataxia and bilateral lower extremities numbness (right greater than left) which had progressed. Additionally, he had experienced an episode of fecal incontinence without any bladder issues. The ataxia had been progressively worsening without significant focal weakness. 

His medical history was significant for ENB of the anterior cranial fossa treated with resection and radiation therapy 13 years prior to this admission. He then underwent left cervical lymph node resection and local radiation of his ENB metastasis 6 years after the initial craniotomy due to the finding of an anterior neck mass. Two years later, during a posttrauma evaluation for neck pain, it was noted on his bone scan that he had multiple metastatic lesions to the cervical spine without any cord compression. At that time, he started on chemotherapy with Cytoxan, adriamycin infusion, and DTIC. After some response in his cervical lymph nodes, he was taken off chemotherapy for a period. Due to progressive adenopathy, the patient started on cisplatin and VP-16 for several months. Once again, his symptoms stabilized. 

Three years prior to this admission, due to a diagnosis of leptomeningeal spread, the patient underwent a repeat craniotomy for resection of his anterior fossa recurrence, followed by whole brain radiation. Following a complaint of mid-lower back pain, he was diagnosed with a T11 lesion and had local radiation. To treat progression of his leptomeningeal disease, he completed three cycles of temodar in the year prior to this admission. His medical history also included scoliosis surgery with Harrington rods 25 years prior to his last admission.

**Figure 1:**
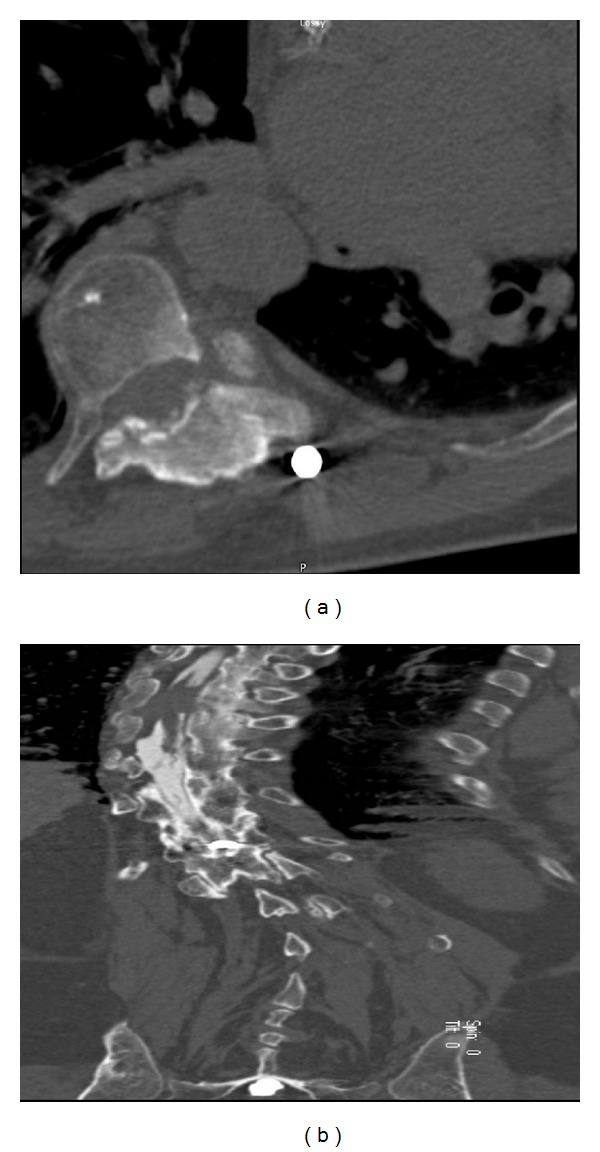
Axial and coronal reformatted CT myelogram images of thoracic spine illustrate compression of spinal cord to the left with complete contrast block at T8-9.

On physical examination, the patient had slight weakness of his left lower extremity, especially his left extensor hallucis longus. He had decreased light touch and pinprick sensation of his right T11 dermatome and diminished proprioception of his left lower extremity. He had decreased rectal tone and hyperreflexia of his left knee with trace right ankle reflexes. There was a positive Babinski sign on the left. Due to artifacts from Harrington rod scoliosis correction of his thoracic spine, we obtained a CT myelogram in addition to the MRI of his thoracic spine. The CT myelogram revealed a near-complete block of contrast at T8-9 (Figure 1). His cervical spine MRI showed stable metastasis to his C5-6 level without any cord compression. Multiple bony lesions throughout the spinal vertebras were noted. 

Given the patient's medical history and symptoms, surgical resection was recommended. He underwent a posterior laminectomy from T7–9. Epidural and intradural extension of the tumor to the ventral left side of the cord with significant cord compression was noted. With a finding of arachnoid plane between the tumor and spinal cord, microsurgical technique was used to remove 80–90% of the tumor and duraplasty was performed from T7–9 to create space in case of tumor recurrence. 

**Figure 2:**
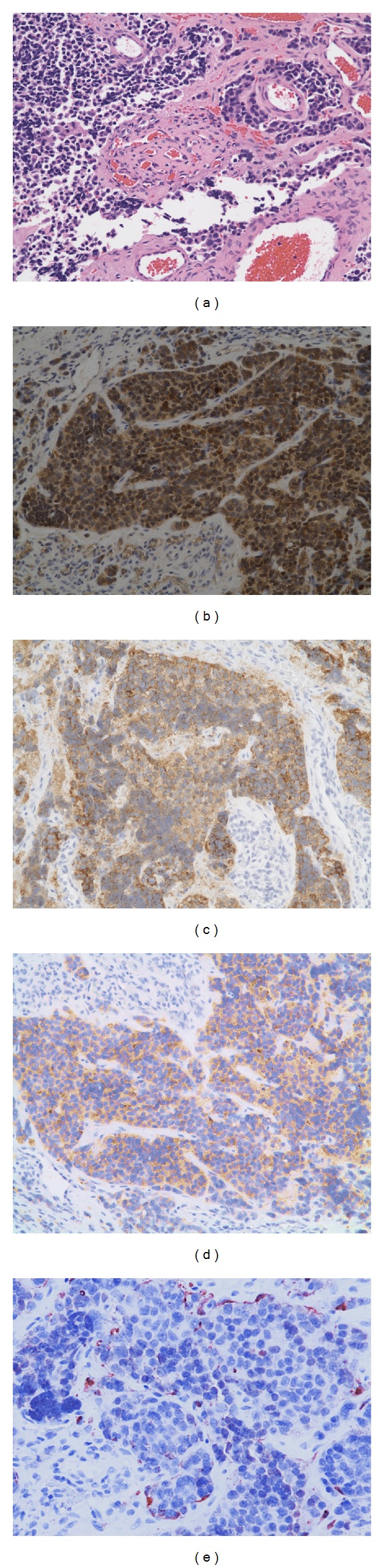
Histologic sections of T8 lesion confirm the preoperative impression of a metastatic esthesioneuroblastoma (a). The tumor is cellular and is positive for markers of neuronal (neuron specific enolase (b), synaptophysin (c), and chromograninn (d)), as well as of a sustentacular differentiation (e). Original magnification: ×200.

Microscopic examination of T8 lesion revealed a small cell tumor lacking necrosis (Figure 2(a)). Immunohistochemical analysis showed diffuse immunoreactivity for neuron-specific enolase and synaptophysin (Figures 2(b) and 2(c)), with scattered cell positive for chromogranin A (Figure 2(d)). S100 stain showed perilobular disposition of flattened cells, indicative of sustentacular differentiation (Figure 2(e)). The findings were of a metastatic neuroendocrine carcinoma, compatible with an esthesioneuroblastoma.

Postoperatively, the patient did well with a stable neurological exam followed by completion of 2 cycles of chemotherapy with Cytoxan, adriamycin, and DTIC. At his follow-up visits every 3 months, his tumor remained stable on CT and MRI studies for a period of one year. 

**Figure 3:**
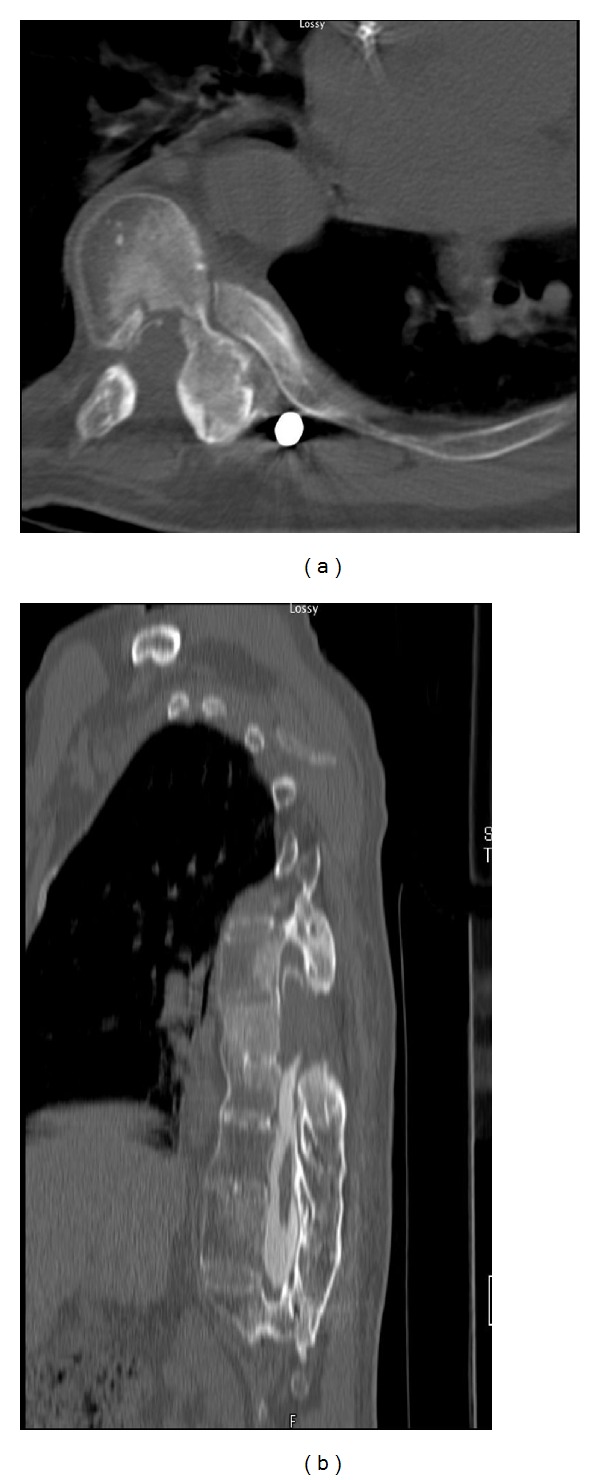
Axial and sagittal reformatted CT myelogram images over a year after the initial resection reveal tumor recurrence at the laminectomy segment with contrast block.

Shortly thereafter, the patient presented with worsening ataxia and bilateral lower extremity numbness and paresthesias. His motor exam was worse indicating bilateral 4/5 strength diffusely in his lower legs. The MRI and CT myelograms of his thoracic spine revealed recurrent tumor extending above the resection cavity from T7-8 with significant cord compression (Figure 3). 

The patient underwent a microsurgical resection of his intradural tumor with extension of his laminectomy. Ventral-lateral extension of his tumor to the neural foramens was noted. The canal was widened and once again, duraplasty from T6–9 was performed. The histology was of a persistent esthesioneuroblastoma. Postoperatively, his exam remained stable and he was discharged to a rehab facility. The patient subsequently had local radiation to his thoracic surgical cavity and his lower cervical spine (C5-6), which showed progression of his tumor without significant clinical symptoms.

**Figure 4:**
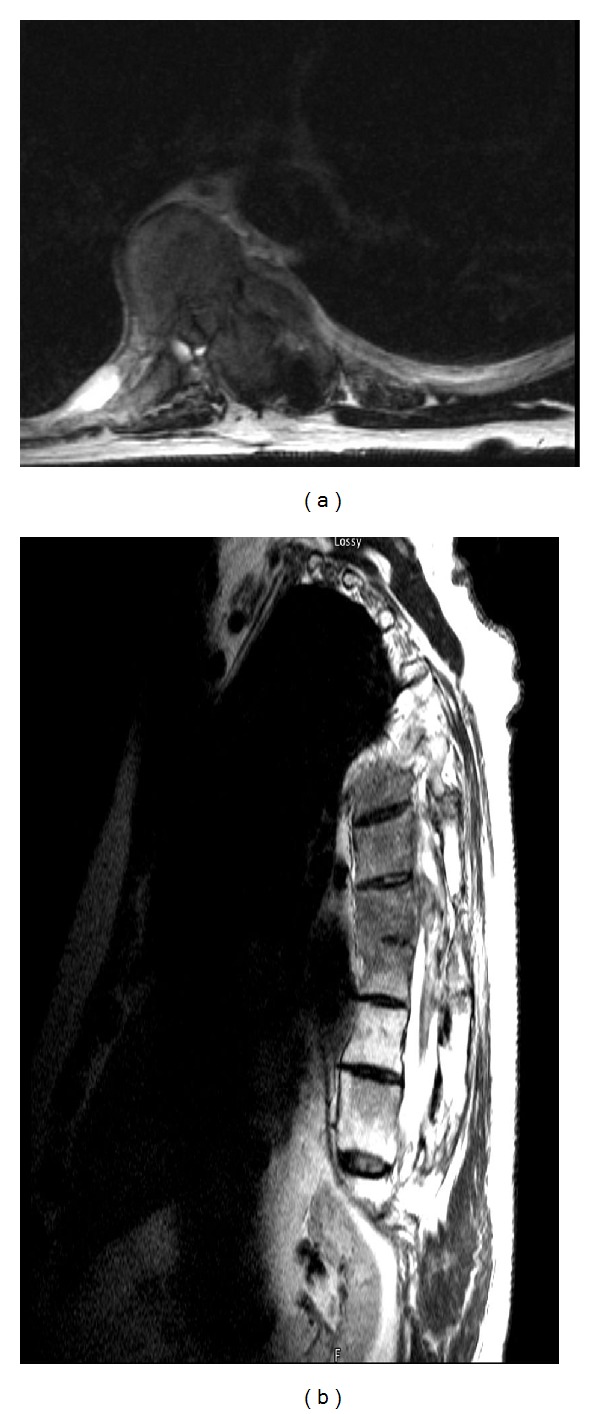
A year after his second resection. Axial and sagittal T2 MRI images of Thoracic spine show anterior lateral tumor recurrence with spinal cord compression to the left. Complete CSF block at T8-9 was noted.

A year later, despite radiotherapy and aggressive treatment, the patient again presented with worsening lower extremities weakness. His MRI and myelograms illustrated recurrence of his tumor in his upper thoracic spine and contrast block at the T8 level (Figure 4). Consequently, the patient underwent staged resection and exploration of his tumor from T4–T10 with decompression of his neoplasm. Onlay duraplasty with subarachnoid drain placement above the resection cavity was performed. He continued treatment with physical therapy and was followed by the pain service for his neurogenic pain and spasm. 

**Figure 5:**
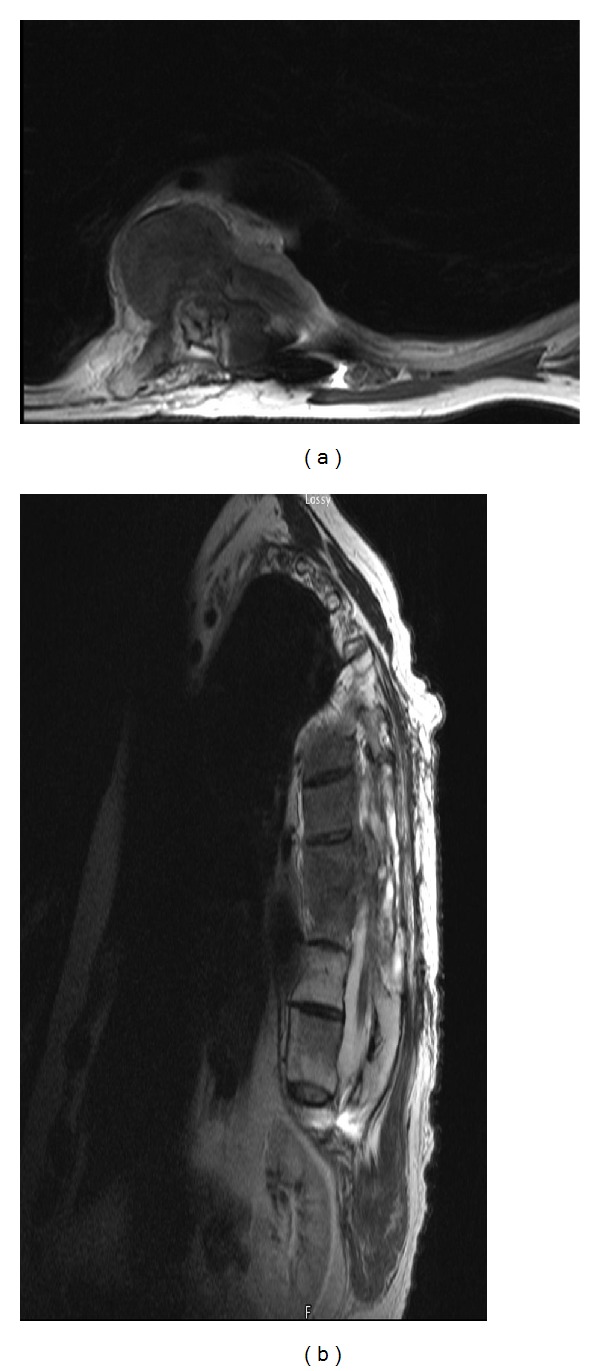
His last axial and sagittal MRI images eight months after his last surgery illustrate aggressive tumor involvement of the thoracic spine with significant cord compression.

Eight months after his last resection, the patient's MRI studies showed progression of his tumor with soft tissue masses throughout his thoracic spine and diffuse gliosis. No further significant canal stenosis was noted (Figure 5). He was dependent on a wheelchair, but physical therapy felt that he might get some improvement in strength with intense rehabiltiation. Thus, he was transferred to inpatient rehabiltiation. Three years later, he passed away from pneumonia.

## 3. Discussion 

Esthesioneuroblastoma is a rare malignant intranasal neoplasm that originates from neural crest cells of the olfactory epithelium. Tumors are normally located high in the nose, above the middle turbinate, and fixed to the cribriform plate or ethmoid sinuses [14]. Local invasion through the paranasal sinuses and orbits is common, but extension into the anterior cranial fossa and distant metastatic disease occurs infrequently [15, 16]. ENB represents 3 percent of intranasal neoplasms.

Berger and Luc first described ENB in France in 1924 [17, 18]. Since 1924, close to 1,000 cases of ENB have been documented in world literature using various names: esthesioneuroepithelioma, esthesioneurocytoma, esthesioneuroblastoma, olfactory esthesioneuroma, intranasal neuroblastoma, and neural olfactory tumor [17]. In 1951, Schall and Lineback described 3 cases of the tumor in the American literature. 

The best treatment modalities for ENB have not been well defined by prospective studies due to the low incidence of tumors [3]. However, case reports and retrospective studies have provided insight into the natural progression of the disease and treatment options. The literature suggests no gender bias, with equal tumor occurrence among males and females [19, 20]. Tumor distribution is bimodal, occurring most frequently in the second and sixth decades of life [3–5]. The two most common presenting symptoms of ENB are nasal obstruction and epistaxis [1, 4, 5]. Other symptoms include proptosis, headache, anosmia, diplopia, rhinorrhea, tearing, eye pain, facial pain, nausea, and facial swelling [1, 5, 15, 17]. The most common physical exam finding is a red friable polyploid mass in the nasal cavity [1, 5]. Additional physical exam findings include exophthalmos and neck mass [4]. 

The tumor is considered to be of neuroectodermal origin and to arise from the olfactory epithelium [1]. ENB is thought to develop from an immature neuroblast or neurocyte [1]. Microscopically, the tumor appears as small round cells forming lobules or diffuse sheets within the fibrovascular stroma [4]. The pathological diagnosis of ENB is classically characterized by the presence of pseudorosettes and true rosettes [4]. Three main histological variants have been described in the literature: olfactory esthesioneuroepithelioma, olfactory esthesioneurocytoma, and olfactory esthesioneuroblastoma. However, the histological subtype has not been shown to correlate with disease outcome or survival [3]. 

Multiple staging systems have been designed to classify ENB. In 1976, Kadish et al. created the first staging system based on clinical grounds, classifying the lesions by anatomic location: Group A, tumor within the nasal cavity; Group B, tumor within the nasal cavity and paranasal sinuses; Group C, extension of tumor outside of the nasal cavity and paranasal sinuses [11]. A modified 14-staging system designed by Foote et al. added a fourth group: Group D for cervical nodal or distant metastases [21]. 

The UCLA staging system developed by Dulguerov et al. classified the tumor based on tumor invasion and related to the level of invasion into four clinical outcomes: T1, tumor involving the nasal cavity and/or paranasal sinuses (excluding the sphenoid), sparing the most superior ethmoidal cells; T2, tumor involving the nasal cavity and/or paranasal sinuses (including the sphenoid) with extension to or erosion of the cribriform plate; T3, tumor extending into the orbit or protruding into the anterior cranial fossa; T4, tumor involving the brain [4]. CT and MRI are used to monitor disease progression. Based on this literature review, we developed a classification system of metastatic ENB to the spine (Table 1).

The metastatic rate of ENB has been estimated to range from 10 to 62 percent [10]. The most common site of metastatic disease is the cervical lymph nodes but metastatic disease has been found in the long bones, parotid, meninges, breast, lung, spinal cord, prostate, abdominal viscera, and pelvis [15]. 

Spinal metastasis is rare. In the literature, twenty-one cases of metastatic ENB to the spine, spinal cord, and spinal meninges have been documented (Table 2). The rate of local recurrence for this tumor after therapy is 57% with a survival rate of 58% at the end of five years [22]. 

Prognostic factors must be considered when determining the best treatment for ENB. Murakami et al. reported that complete surgical resection and histological tumor grade are the two most important prognostic factors [23]. To improve prognosis, Hwang et al. emphasized the importance of diagnosing the disease in an early stage and regular radiologic followups to detect disease recurrence [4]. A definitive treatment plan for ENB has not been outlined in the literature due to rare incidence of disease, difficulty in establishing the diagnosis, and lack of randomized control trials to study treatment. However, a thorough review of the literature states that surgical resection is the primary form of treatment and can be used alone or in combination with radiation therapy. 

In a retrospective study by Broich et al., the treatment and outcomes for 898 patients with ENB were reviewed [17]. Patients received surgery, radiation therapy, a combination of surgery and radiation therapy, chemotherapy, or bone marrow transplant [17]. The results showed survival rates to be the highest among patients who received a combination of surgery and radiation therapy [17]. 

In a study of 49 patients with ENB by Morita et al., 38 patients who underwent gross tumor resection with or without radiation therapy had better survival and disease-free periods than patients who had partial tumor resection or biopsy with radiation therapy [24].

In a study by Elkon et al., the best treatment modality was determined based on Kadish Stage [3]. For patients with Stage A or B disease, surgery or radiation therapy alone was as beneficial as combination therapy for the treatment of disease [3]. In patients with Stage C disease, combined treatment appeared to be the best form of therapy and gave patients the greatest chance for cure [3]. Thus, the literature suggests that the best initial treatment plan for ENB is surgical resection of the tumor with or without radiation therapy. 

The role of chemotherapy in treatment of ENB is not clearly defined but the literature suggests the use of chemotherapy for palliation in advanced disease or recurrent disease [7]. ENB is responsive to multiple chemotherapeutic agents: cyclophosphamide, vincristine, triethylenethiophosphoramide, doxorubicin, dacarbazine, nitrogen mustard, and a combination treatment with etoposide, ifosfamide, and cisplatin [25]. However, no definitive chemotherapy treatment has been defined in the literature. 

In a 2004 study by Kim et al., nine out of eleven patients treated with a combination therapy of etoposide, ifosfamide, and cisplatin had a treatment response [25]. There were 2 complete responses and 7 partial responses to treatment. However, the treatment response did not come without toxic side effects: grade 3/4 neutropenia occurred after 37% of chemotherapy cycles, and febrile neutropenia occurred after 2 cycles of chemotherapy. 

Wade et al. reported the response to chemotherapy in five patients with Kadish Stage B or C disease and eight cases of ENB treated with chemotherapy [26]. Complete and partial remission occurred in 8 out of 13 patients treated with cyclophosphamide, vincristine, nitrogen mustard, and thiopeta [26].

In a 2008 study by Kim et al., expression of the bcl-2 tumor marker in ENB patients suggests better response to chemotherapy [27]. One of the patients in the study with diffuse bcl-2 expression had complete remission after treatment with neoadjuvant chemotherapy [27]. A patient without bcl-2 expression had no response to neoadjuvant chemotherapy [27]. Patients with partial bcl-2 expression had partial response to neoadjuvant chemotherapy [27]. Thus, tumor markers may play an important role in the development of chemotherapeutic treatment options for ENB. Further clinical trials must be carried out in order to find the most effective agent for treatment of this tumor. 

Definitive treatment for metastatic ENB to the spine has not been well defined due to the scarcity of presentation and only twenty-eight cases were documented in the literature. Of the twenty-one patients, thirteen are dead, four are living, and the outcome of four patients is unknown (Table 2). 

In one case of metastatic ENB to the spine, described by Rao et al. [22], the patient was treated with surgical resection of spinal lesions in the cervical, thoracic, and lumbar spine [23]. The patient was then treated with six cycles of combination chemotherapy: carboplatin, etoposide, and cyclophosphamide over 6 months [22]. Follow-up MRI of the spine 12 months after chemotherapy showed stabilization of the size of the lesions or a small increase in their size [22]. From a clinical standpoint, the patient improved neurologically with both increased strength and intact sensation in the upper extremities [22]; however, the patient suffered side effects from the chemotherapy: anemia, fatigue, hypotension, and lightheadedness with the possibility of adrenal insufficiency from taking dexamethasone [22]. The patient is reported to be doing well clinically with relief of symptoms. This case demonstrates that while the combination of surgery and chemotherapy can help to reduce symptoms and extend life expectancy, this symptom relief and extended life span come with a cost—the side effects from chemotherapy. 

In a different case of metastatic ENB to the spine, described by Arnold et al. [15], treatment consisted of surgical resection of the thoracic spinal lesion along with combined Temodar chemotherapy and radiation therapy to the spine [15]. The follow-up MRI one year after surgery showed no tumor recurrence and the patient has had no thoracic spine recurrence two years after operation [15]. 

## 4. Conclusion

These case reports suggest that a combination of surgical resection, chemotherapy, and radiation therapy can be used to provide symptom relief, prolong life, and help to prevent recurrence of disease. A definitive treatment for metastatic EBN to the spine is needed, however, because thirteen of the 21 patients documented in the literature died and did not respond to these treatments. Perhaps, the expression of a genetic tumor marker, such as bcl-2, may hold the key to a cure. Further research is needed to outline a definitive treatment option for esthesioneuroblastoma.

## Figures and Tables

**Table 1:** Cedars-Sinai classification system for metastatic esthesioneuroblastoma to the spine.

Stage	Anatomic location of lesion
CS0-leptomeningeal disease	Involvement of the CSF and leptomeninges (arachnoid and pia mater)
CS1a-one level disease	Involves one level of the spine or spinal cord: cervical, thoracic, lumbar, or sacral
CS1b-one level disease and leptomeningeal disease	Involves one level of the spine or spinal cord: cervical, thoracic, lumbar, or sacral; with involvement of the CSF and leptomeninges (arachnoid and pia mater)
CS2a-two level disease	Involves two levels of the spine or spinal cord: cervical, thoracic, lumbar, or sacral
CS2b-two level disease and leptomeningeal disease	Involves two levels of the spine or spinal cord: cervical, thoracic, lumbar, or sacral; with involvement of the CSF and leptomeninges (arachnoid and pia mater)
CS3a-three level disease	Involves three levels of the spine or spinal cord: cervical, thoracic, lumbar, or sacral
CS3b-three level disease and leptomeningeal disease	Involves three levels of the spine or spinal cord: cervical, thoracic, lumbar, or sacral; with involvement of the CSF and leptomeninges (arachnoid and pia mater)
CS4a-four level disease	Involves four levels of the spine or spinal cord: cervical, thoracic, lumbar, or sacral
CS4b-four level disease and leptomeningeal disease	Involves four levels of the spine or spinal cord: cervical, thoracic, lumbar, or sacral; with involvement of the CSF and leptomeninges (arachnoid and pia mater)

**Table 2:** Summary of location or primary and spinal metastates, initial treatment, and outcome in previously published cases of metastatic esthesioneuroblastoma.

Reference	Age (yrs) sex	Location of primary	Initial treatment	Recurrence and presenting spinal symptoms	Location of spine metastases	Outcome
[28]	67 M	Nasal cavity	Biopsy, radiation therapy (total dose 6000 r)	Sacral and coccygeal pain with sphincter incontinence	lumbar spine (cauda equina)	Death
[1]	NA	Nasal cavity	Surgery, chemotherapy, and radiation therapy	NA	Spinal cord	NA
[3]	NA	Nasal cavity	Surgery and radiation therapy	NA	Spinal cord	Death
[29]	47 M	Nasal cavity, frontal lobe	Biopsy and radiation Therapy	NA	lumbar spine	Death
[5]	39 M	Nasal cavity	Surgery (resection)	NA	Spinal cord and vertebral column	Death
[30]	75 F	Nasal cavity	Biopsy, radiation therapy, and chemotherapy	Radiating pain in the left leg, decreasing urinary continence	Lumbar spine (cauda equina); malignant tumor cells in CSF	Death
[20]	20 F	Nasal cavity.	Surgical resection; radiation therapy (plus WBRT); systemic chemotherapy.	Left arm pain and numbness with mild weakness and diminished reflexes	Cervical 6 nerve foramen; malignant tumor cells in CSF	Death
[24]	NA	Nasal cavity	Surgical resection, radiation therapy, and chemotherapy	NA	Epidural spine	NA
[10]	76 F	Nasal cavity	Biopsy and radiation therapy	Pain over the buttock with radiation to thigh with paresthesias in soles of the feet	lumbar spine (cauda equina); malignant tumor cells in CSF	Death
[31]	35 M	Nasal cavity	Surgical resection	NA	Thoracic 5–8	Death
[4]	NA	NA	Surgical resection and/or radiation therapy and/or chemotherapy	NA	Thoracic vertebrae	Death
[7]	51 F 47 F	Nasal cavity	(1): Subtotal resection and radiation therapy (2): Gross total resection and radiation therapy	Thoracic pain and lower extremity weakness	Thoracic 8 subarachnoid nodule and lumbar spine	Death
[16]	NA	NA	Radiation therapy, surgical resection, chemotherapy, or combination	NA	Intramedullary spinal cord	NA
[19]	28 M	Lamina cribrosa	Debulking surgery and radiation therapy	Pain between the scapulae	Thoracic 4–8	Living
[23]	37 M	Nasal cavity, frontal lobe	Surgical resection and radiation therapy	Poor visual acuity and diplopia	Cervical 3–7 and lumbar 2–4	Death
[12]	49 M	Nasal cavity	Surgical resection	Low back pain	Lumbar spine (cauda equina)	Living
[32]	51 F	Kadish Stage C ENB	Surgical resection and radiation therapy	Dyspnea and a painful chest wall mass	Thoracic and lumbar vertebrae	Death
[33]	NA	Nasal cavity	Surgical resection and radiotherapy	NA	Lumbar spine (cauda equina)	Death
[15]	64 M	Head and neck	Surgical resection and radiation therapy	Mid back pain and consistent burning intensity	Thoracic 4–8	Living
[22]	59 M	Nasal cavity, frontal lobe	Surgical resection and radiation therapy	Upper extremity weakness: hand clumsiness, tingling of the arms, and upper chest	Metastases to the cervical, thoracic, and lumbar spinal cord	Living
